# Deletion of ASK1 Protects against Hyperoxia-Induced Acute Lung Injury

**DOI:** 10.1371/journal.pone.0147652

**Published:** 2016-01-25

**Authors:** Jutaro Fukumoto, Ruan Cox, Itsuko Fukumoto, Young Cho, Prasanna Tamarapu Parthasarathy, Lakshmi Galam, Richard F. Lockey, Narasaiah Kolliputi

**Affiliations:** 1 Division of Allergy and Immunology, Department of Internal Medicine, Morsani College of Medicine, University of South Florida, Tampa, Florida, United States of America; 2 Department of Molecular Medicine, Morsani College of Medicine, University of South Florida, Tampa, Florida, United States of America; University of Illinois College of Medicine, UNITED STATES

## Abstract

Apoptosis signal-regulating kinase 1 (ASK1), a member of the MAPK kinase kinase kinase (MAP3K) family, is activated by various stimuli, which include oxidative stress, endoplasmic reticulum (ER) stress, calcium influx, DNA damage-inducing agents and receptor-mediated signaling through tumor necrosis factor receptor (TNFR). Inspiration of a high concentration of oxygen is a palliative therapy which counteracts hypoxemia caused by acute lung injury (ALI)-induced pulmonary edema. However, animal experiments so far have shown that hyperoxia itself could exacerbate ALI through reactive oxygen species (ROS). Our previous data indicates that ASK1 plays a pivotal role in hyperoxia-induced acute lung injury (HALI). However, it is unclear whether or not deletion of ASK1 *in vivo* protects against HALI. In this study, we investigated whether ASK1 deletion would lead to attenuation of HALI. Our results show that ASK1 deletion *in vivo* significantly suppresses hyperoxia-induced elevation of inflammatory cytokines (i.e. IL-1β and TNF-α), cell apoptosis in the lung, and recruitment of immune cells. In summary, the results from the study suggest that deletion of ASK1 in mice significantly inhibits hyperoxic lung injury.

## Introduction

Acute lung injury (ALI) is a major clinical issue in the United States and is characterized by diffuse alveolar damage due to acute inflammation [1–3]. ALI-associated inflammatory response includes elevation of proinflammatory cytokines, immune cell infiltration, and pulmonary edema. High concentration oxygen therapy is an essential therapeutic remedy to offset hypoxemia caused by cardiovascular and pulmonary diseases such as ALI [4]. However, prolonged delivery of high concentrations of oxygen is suspected to exacerbate ALI in critically ill patients [5]. Additionally, animal experiments so far have shown that hyperoxia itself could trigger pathological conditions similar to ALI [6]. Thus, hyperoxia-induced acute lung injury (HALI) has been one of the most widely used animal models for studying ALI. There are several ALI models that have been widely used including HALI. Ventilator-induced ALI model has been another major tool to study the pathogenesis of ALI even though it requires large tidal volumes to evoke clinically relevant ALI [7]. While different animal models indicate the involvement of oxidative stress in ALI pathogenesis, the precise molecular mechanisms by which oxidative stress activates lung inflammation is not fully elucidated.

Apoptosis signal-regulating kinase-1 (ASK1) is a member of the mitogen-activated protein kinase kinase kinase (MAPKs) group and is necessary for ROS induced cell death and inflammation [8]. ASK1 is prompted by many types of stimuli which include oxidative stress, calcium influx, endoplasmic reticulum (ER) stress, DNA damage-inducing agents, and receptor-mediated signaling through tumor necrosis factor receptor (TNFR) [8]. Recent studies suggest the possibility that inhibitors of ASK1 have potential benefits in the management of ALI [9,10] and that ASK1 is considerably activated and involved in HALI [11]. ASK1-deficient cells were also shown to have decreased inflammatory response through reduced activation and production of proinflammatory cytokines such as interleukin (IL)-1β, IL-6, and TNF-α [12]. Although these studies indicate the involvement of ASK1 in oxidative and cellular stress, the overall exploration of ASK1 in lungs and HALI has been minute. In our previous investigation, data indicates that ASK1 expression plays an important role in HALI [13]. However, it is unclear whether or not deletion of ASK1 *in vivo* protects against HALI. Therefore, in this study, we investigated whether ASK1 deletion would lead to attenuation of HALI by assessment of apoptosis in lung epithelial cells as well as immune cells in airspace, immune cell recruitment, and levels of pro-inflammatory cytokines.

## Results

### Hyperoxia-induced lung epithelial apoptosis is attenuated in ASK1 KO mice

**Fig 1:**
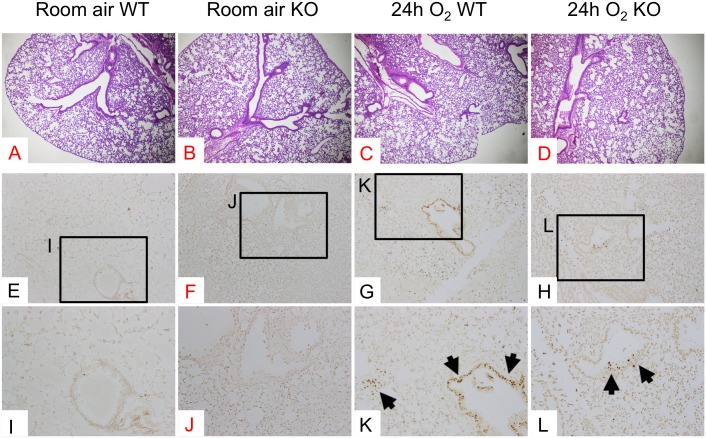
Hyperoxia-induced apoptosis of lung epithelial cells is attenuated in ASK1 KO mice. WT and ASK1 KO mice (n = 4 per each group) were exposed to room air or 100% O_2_ for 24 h. Whole lungs were formalin-fixed, paraffin-embedded and thin sliced sections were subjected to H&E or TUNEL staining. Representative photomicrographs of H&E-stained (**A-D**) and TUNEL-stained (**E-L**) lung tissue sections are shown. Labeled boxes (**I-L**) correspond to their respective enlarged images. Arrows denote brown signals or fragmented DNA, indicating cells undergoing apoptosis. Magnification (A-D): 40X; (E-H): 100X; (I-L): 200X.

To evaluate the effect of ASK1 deletion on histopathological findings of the mouse lungs exposed to hyperoxia, we evaluated H&E-stained lung sections. WT lungs exposed to 24 h of hyperoxia exhibit a slight thickening of bronchiolar epithelium compared to normoxic lungs (Fig 1A & 1C). This hyperoxia-induced morphological alteration was also seen in ASK1 KO lungs (Fig 1B & 1D) and there was no significant difference between hyperoxic WT and ASK1 KO lungs regarding the histopathological severity (Fig 1C & 1D). Histopathological evaluation of H&E-stained lung sections was performed on WT and ASK1 KO mice exposed to 48–72 h of hyperoxia. However, no significant difference was found between WT and ASK1 lungs either (data not shown).

Lung epithelial cell apoptosis and necrosis is one of the key features of hyperoxia-induced lung injury [14,15]. ASK1 is induced by hyperoxic insult, and this induction is a key event in hyperoxia-induced apoptosis [11]. To assess the direct effect of ASK1 deletion on cellular apoptosis in the lung, we detected DNA fragmentation by TUNEL staining using tissue sections from WT and ASK1 KO mice exposed to 24 h of hyperoxia, as well as WT and ASK1 KO room air controls. The results show that cell apoptosis in lung epithelium was attenuated in ASK1 KO mice (arrows in Fig 1L) compared to WT controls (arrows in Fig 1K). These results suggest that ASK1 plays a critical role in hyperoxia-induced lung epithelial apoptosis, which is consistent with previous reports [7]. Note that the protective role of ASK1 against hyperoxia-induced cell apoptosis in the lung tissue as assessed by TUNEL staining was most remarkable at 24 h of hyperoxia, but not at late time points (data not shown). These apparently inconsistent results can be reconciled by the explanations that (1) cells undergoing apoptosis in most tissues are immediately cleared by phagocytes and become histologically undetectable within 1–2 h [16] and that (2) different types of cell death including apoptosis and necrosis occurs in lung epithelial cells under prolonged hyperoxic insult [15]. Our studies utilizing TUNEL assay coupled with morphological analysis using transmission electron microscopy (TEM) indicate that apoptosis is not predominantly seen in alveolar epithelium of the lungs from mice exposed to prolonged hyperoxia (data not shown).

### Deficiency of ASK1 in mice inhibits hyperoxia-induced alteration and increase of apoptotic macrophages

**Fig 2:**
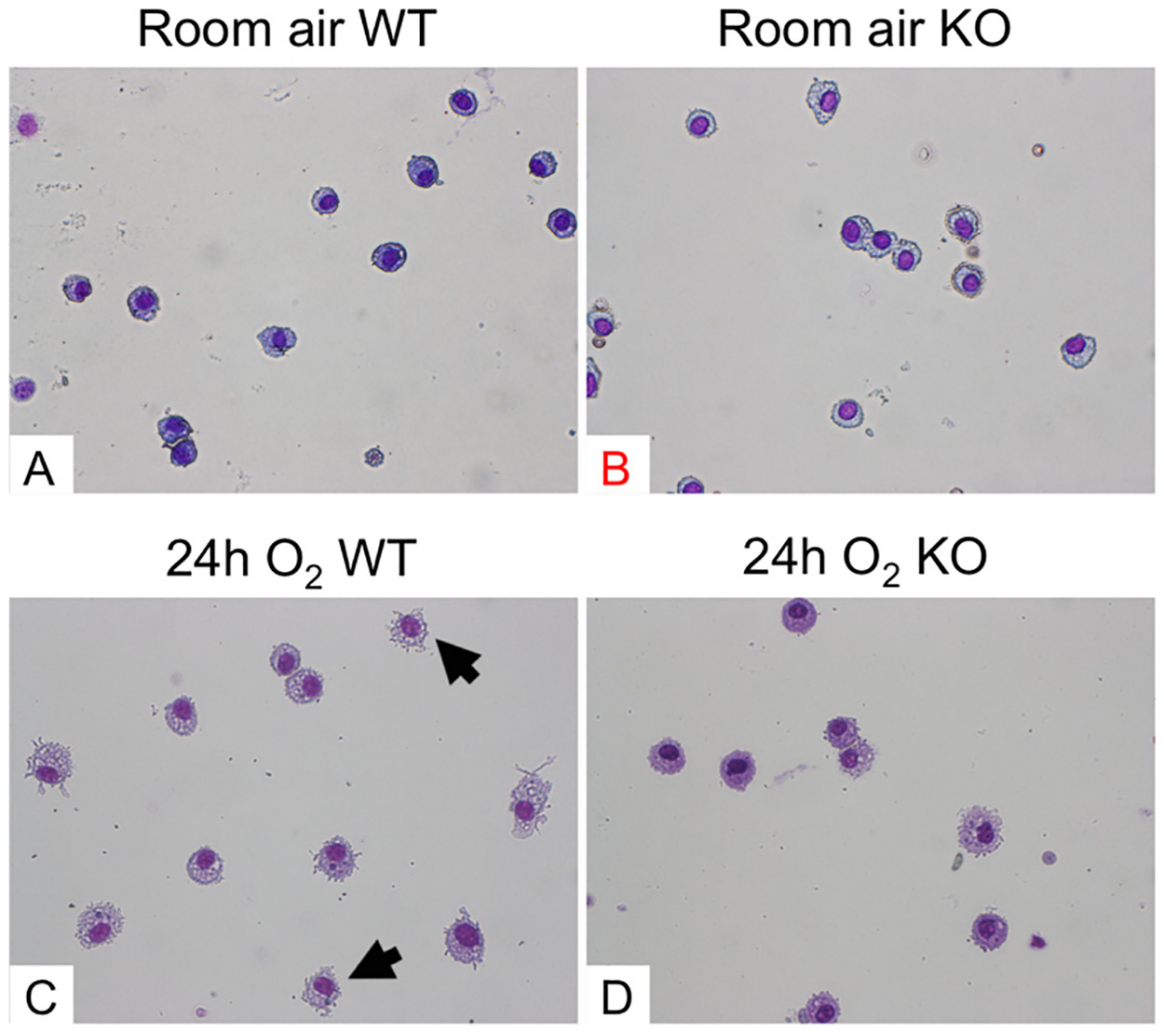
Hyperoxia-induced morphological alteration of alveolar macrophages is inhibited in ASK1 KO mice. Bronchoalveolar lavage (BAL) fluid was collected from WT and ASK1 KO mice (n = 4 per each group) exposed to room air or 100% O_2_ for 24 h (**A-C**). Photomicrographs show representative images of Diff-Quik stained BAL fluid cells. All cells shown here are mononuclear and have a relatively large cytoplasmic space, suggesting that these cells are macrophages or macrophage-committed mononuclear cells that were recruited to the lung airspace. Arrows denote macrophages with shrinking of cytoplasm. Magnification (A, B, & C): 400X.

**Fig 3:**
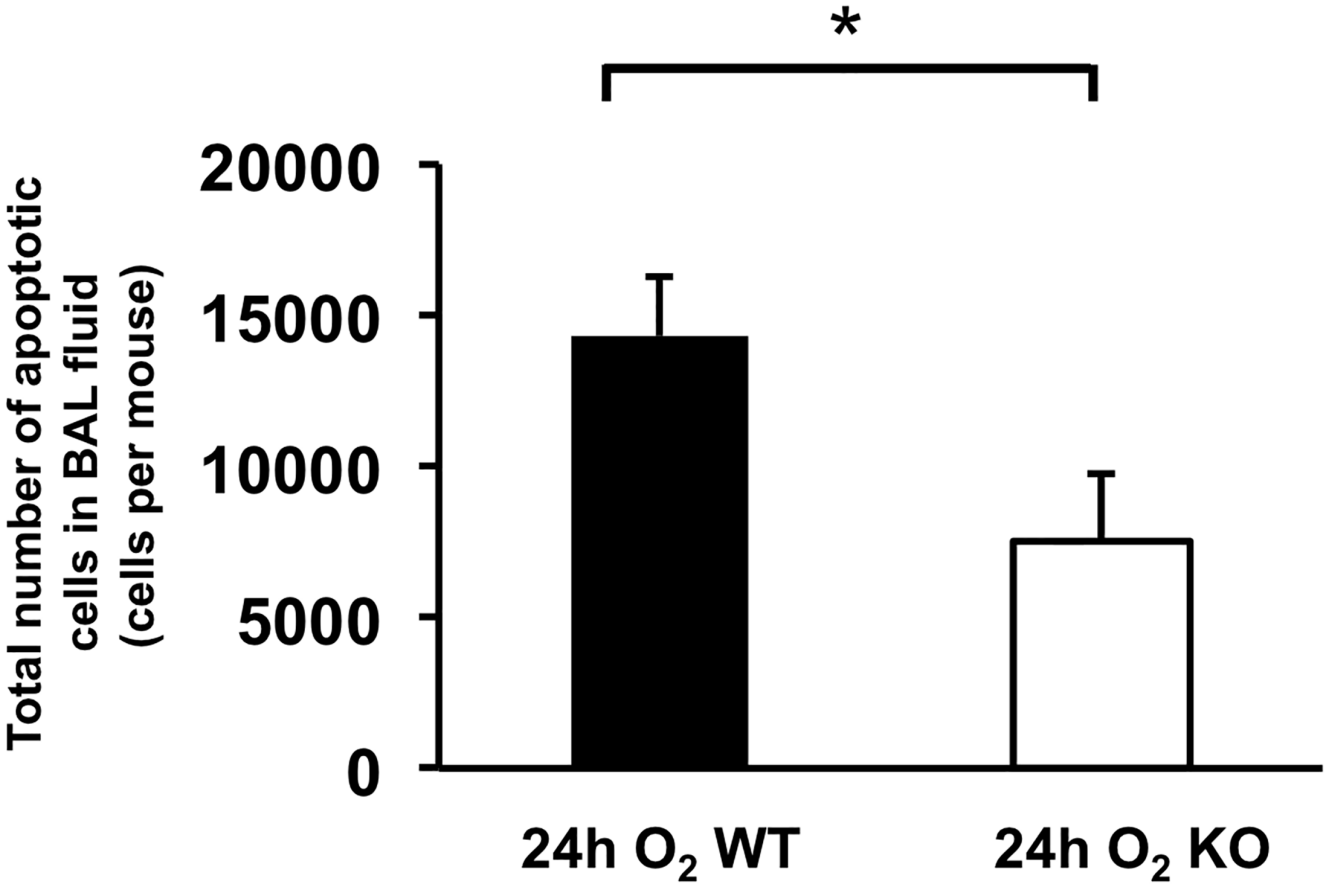
Hyperoxia-induced accumulation of apoptotic macrophages in lung airspace is suppressed in ASK1 KO mice. WT and ASK1 KO mice were exposed to 100% O_2_ for 24 h. Cells in BAL fluid were resuspended in 1mL PBS. The density of apoptotic cells in BAL fluid was evaluated by multiplying the ratio of apoptotic cells and the density of total white blood cells in BAL fluid (details are described in the Materials and Methods section). This was determined by TUNEL and DAPI staining of BAL fluid cells, followed by counting under fluorescent microscope. Results (means ± SEM; n = 6 in each group) are cumulative data of two independent experiments. * p < 0.05.

The influence of ASK1 deletion on alveolar macrophages under hyperoxic condition is not fully understood, especially their viability and/or apoptosis. We investigated the effects of ASK1 deletion on morphology and apoptosis of alveolar macrophages using WT and ASK1 KO mice exposed to 24h of hyperoxia. We chose this early time point because 24h was the optimized length of hyperoxic exposure in which we could distinguish morphological alteration of macrophages, and yet cytokine elevation and recruitment of other immune cell types in the airspace was minimal. Thus, it was simple to assess the immediate effects of ASK1 deletion on the behaviors of alveolar macrophages under hyperoxic insult. Diff-Quik stained BAL fluid cells from WT mice exposed to 24h of hyperoxia showed predominance of relatively large and round mononuclear cells which are considered to be relatively intact macrophages (Fig 2C). Meanwhile, some cells displayed abnormal appearance with shrinking of cytoplasm, a morphological feature of apoptotic cells (arrows in Fig 2C). This morphological abnormality was more apparent in the BAL fluid cells of WT mice exposed to longer periods of hyperoxia (data not shown). Remarkably, BAL fluid cells from ASK1 KO mice under hyperoxia displayed lesser degrees of morphological abnormality compared to WT mice (Fig 2D). These results suggest that ASK1 deletion reduces the sensitivity of alveolar macrophages to hyperoxic insult. Based on these results, we hypothesized that ASK1 KO-associated suppression of hyperoxia-induced morphological transition of alveolar macrophages accompanies suppressed apoptosis. To test this hypothesis, the densities of apoptotic cells in lung airspace were evaluated by finding the product of the cell density of BAL fluid and ratio of apoptotic to total cells, which was determined by combined TUNEL and DAPI staining of formalin-fixed BAL fluid cells followed by counting under fluorescent microscope. The total number of apoptotic cells in lung air space of ASK1 KO mice lung exposed to 24h of hyperoxia was significantly decreased compared to WT hyperoxic controls (Fig 3). Results from Diff-Quik-stained BAL fluid cells suggest that ASK1 deficient macrophages are less susceptible to hyperoxia-induced apoptosis than WT controls.

### Hyperoxia-induced immune cell recruitment and inflammatory cytokine elevation in the lung is suppressed in ASK1 KO mice

**Fig 4:**
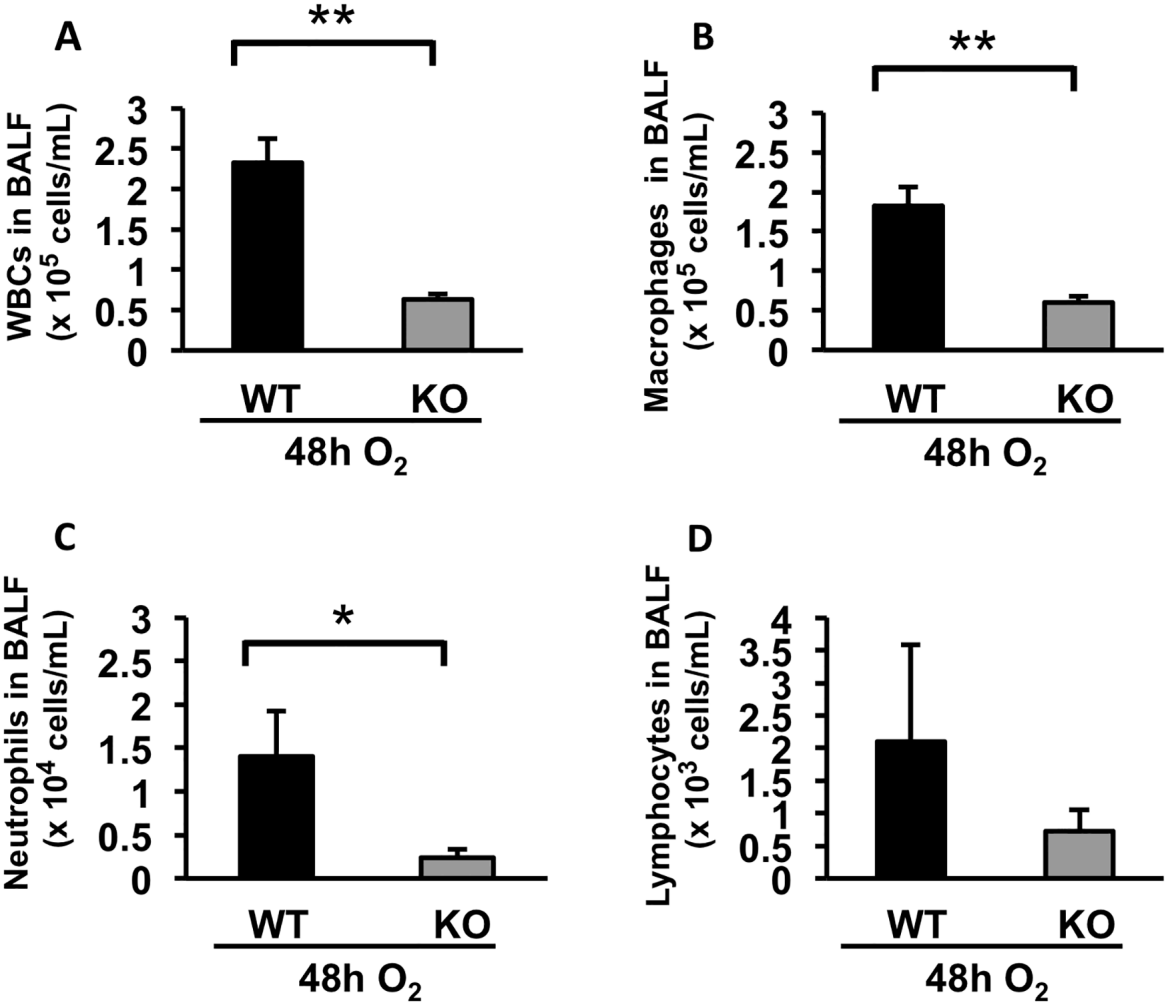
Hyperoxia-induced recruitment of immune cells to the lung airspace is attenuated in ASK1 KO mice. WT and ASK1 KO mice were exposed to 100% O_2_ for 48 h. Cells in BAL fluid were resuspended in 1mL PBS. The density of total white blood cells **(A)**, macrophages **(B)**, neutrophils **(C)**, and lymphocytes **(D)** were determined by total cell count using a hemocytometer and differential cell count using Diff-Quik-stained cells. Results (means ± SEM; n = 4 in each group) are shown. The noted experiments are representative of a minimum of two similar evaluations. * p < 0.05; ** p < 0.01.

**Fig 5:**
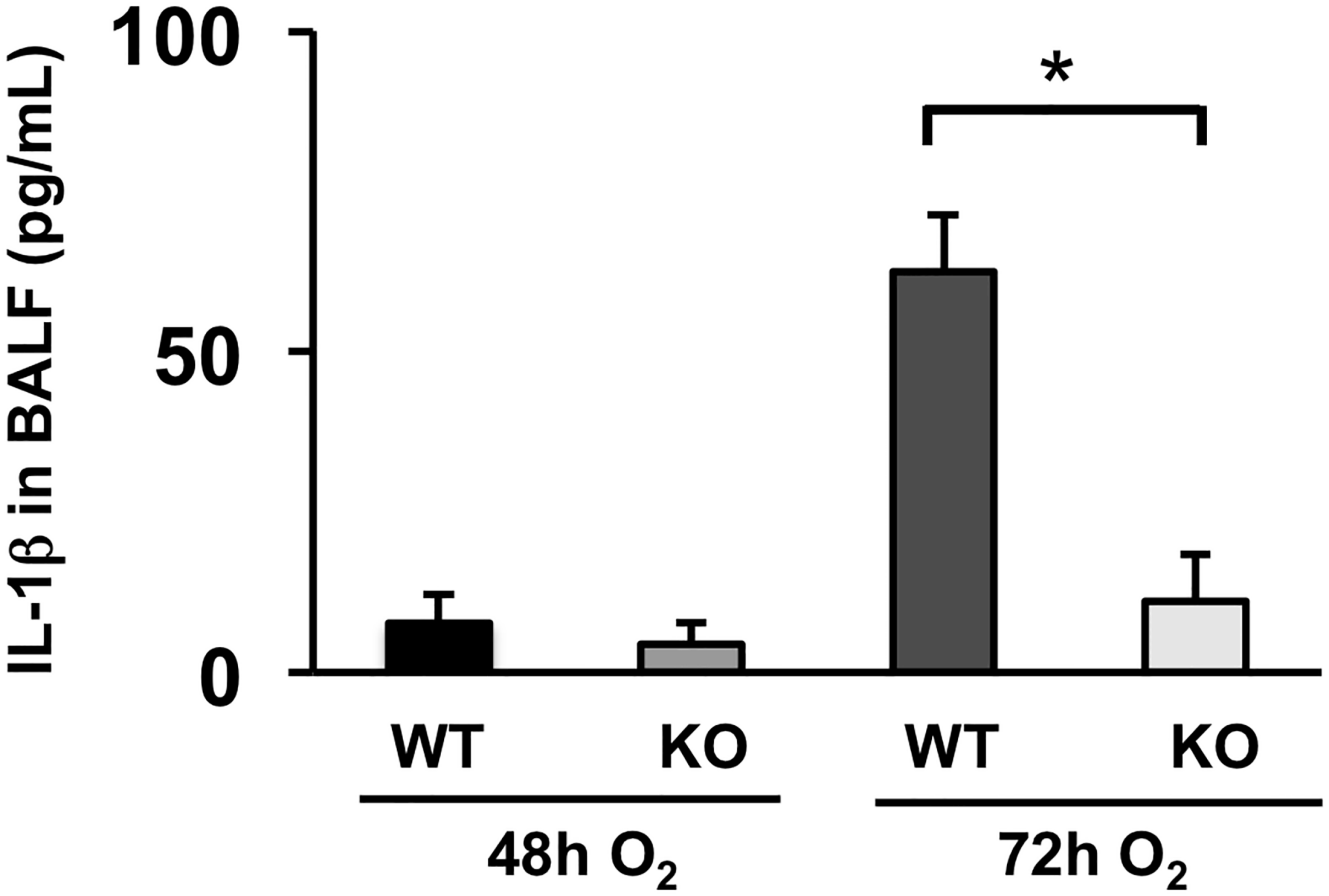
Hyperoxia-induced IL-1β elevation in the lung airspace is suppressed in ASK1 KO mice. WT and ASK1 KO mice were exposed to 100% O_2_ for 48 or 72 h. The concentrations of IL-1β (pg/mL) in supernatants of BAL fluid were determined using ELISA. Results (means ± SEM; n = 4 in each group) are shown. The noted experiments are representative of a minimum of two similar evaluations. * p < 0.05.

**Fig 6:**
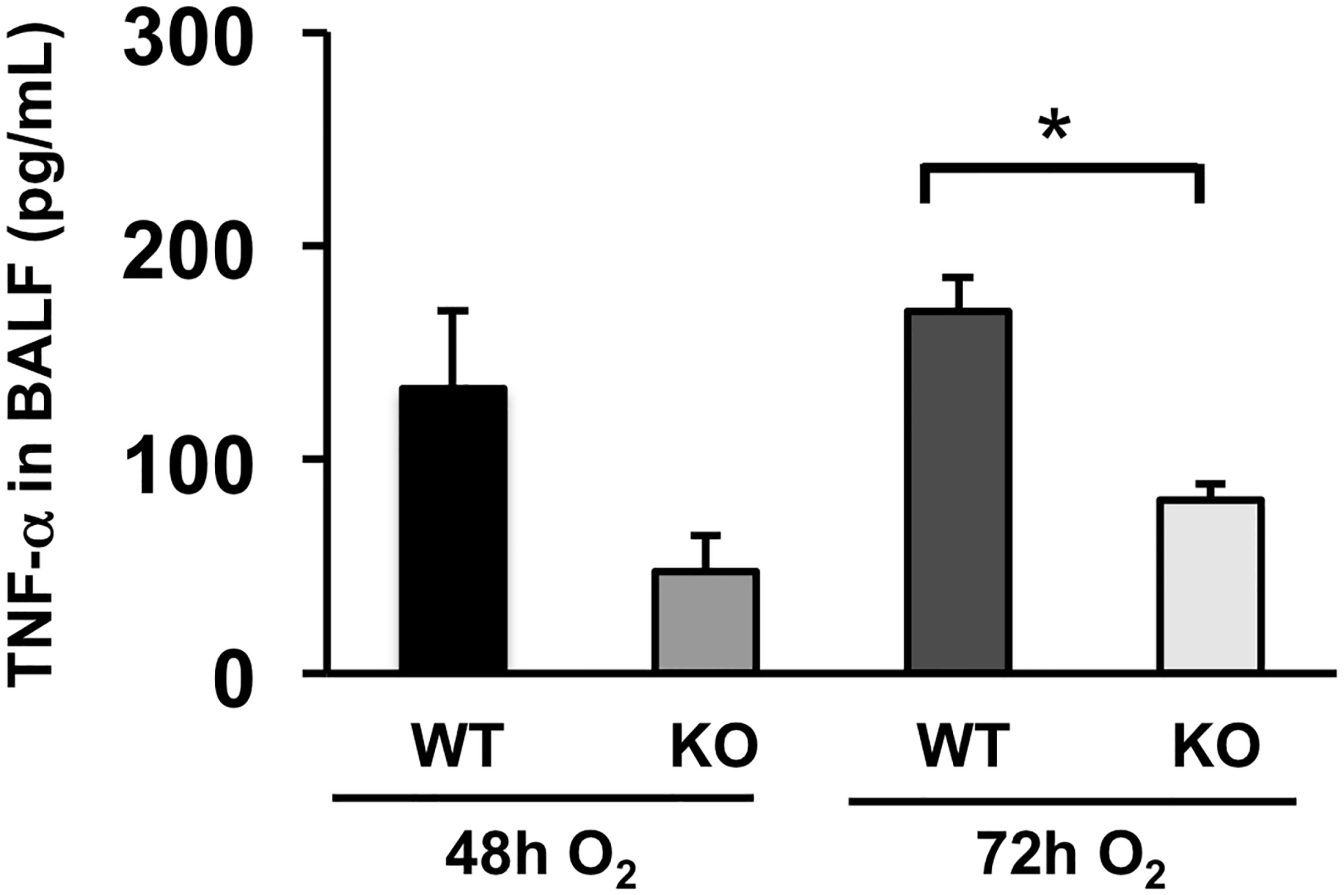
Hyperoxia-induced TNF-α elevation in the lung airspace is suppressed in ASK1 KO mice. WT and ASK1 KO mice were exposed to 100% O_2_ for 48 or 72 h. The concentrations of TNF-α (pg/mL) in supernatants of BAL fluid were determined using ELISA. Results (means ± SEM; n = 4–6 in each group) are shown. The noted experiments are representative of a minimum of two similar evaluations. * p < 0.05.

The extent of immune cell infiltration to the lung airspace is one of the most reliable indexes to evaluate the severity of ALI. We quantified the number of immune cells in BAL fluid at a 48h time point. The results show that each density of total immune cell, macrophages, neutrophils, and lymphocytes, in BAL fluid is significantly decreased in ASK1 KO mice compared to WT controls (Fig 4A–4D & S1 Table). IL-1β is a cytokine known to have the most potent effect in the lungs of early ALI patients [17,18]. Secretion of this cytokine can exacerbate the inflammatory response by inducing a myriad of other proinflammatory cytokines [19]. We analyzed the levels of IL-1β in bronchoalveolar lavage (BAL) fluid of WT and ASK1 KO mice exposed to hyperoxia for 48 and 72 hours. The results show that hyperoxia-induced oxidative stress increased the level of IL-1β in lung airspace, yet was significantly hampered in ASK1 KO mice with an 82% decrease compared to WT controls at the 72h time-point (Fig 5 & S1 Table). TNF-α is the most potent proinflammatory cytokine member of the TNF super family and is recognized as a mediator of the pulmonary inflammatory response [20]. We analyzed the levels of TNF-α in BAL fluid of WT and ASK1 KO mice exposed to hyperoxia for 48 and 72 hours. The results show that hyperoxia-induced oxidative stress increased the level of TNF-α in lung airspace, but was significantly reduced in ASK1 KO mice with a 52% decrease compared to WT controls at the 72h time-point (Fig 6 & S1 Table). Based on our previous research, cytokine elevation and immune cell recruitment is best evaluated at late timepoints with respect to ASK1 deletion. These results suggest that ASK1 has a critical role in the pathogenesis of HALI.

## Discussion

ASK1 is involved in ROS-induced apoptosis and inflammation [21]. Our recent studies suggest that ASK1 is significantly activated and involved in HALI [13]. In addition, we have also shown that SOCS-1 protection against HALI is associated with suppression of ASK1 expression [11]. However, it is not well understood whether or not deletion of ASK1 protects against oxygen toxicity *in vivo*. The purpose of this study is to provide *in vivo* evidence to expedite the development of ASK1 therapeutic targets. The present study shows that (i) ASK1 deletion attenuates hyperoxia-induced ALI and confers protection to mice exposed to hyperoxia; (ii) ASK1 deficiency suppresses hyperoxia-induced cell apoptosis in the lung; (iii) hyperoxia-induced immune cell infiltration was reduced in mice lacking ASK1; and (iv) ASK1 deletion suppressed hyperoxia-induced elevation of inflammatory cytokines.

Several *in vitro* and *in vivo* studies have demonstrated that ASK1 acts as a proapoptotic and proinflammatory molecule in oxidative stress related diseases [11,22]. ASK1 is also known to play an important role in oxidative stress-induced cell death [23]. Once ASK1 becomes activated by dissociation of Thioredoxin (Trx), an important cellular and mitochondrial antioxidant, ASK1 can then induce cell death through the MAPK signaling pathway [23]. In addition, it has been documented that ASK1-deficient cardiomyocytes are protective against hydrogen peroxide (H_2_O_2_)- or calcium-induced cell death [24]. These studies suggest that ASK1 has a pivotal role in mediating various mechanisms of oxidative-stress induced cellular dysfunction and death, while the absence of ASK1 is protective.

It is well established that epithelial cell death plays an important role in HALI [25,26]. In the lung epithelium, oxygen toxicity is known to trigger various forms of cell death including the induction of apoptosis [6,15]. Our results indicate that ASK1 deletion hampers hyperoxia-induced apoptosis of lung epithelial cells, which is in agreement with recent studies [11]. Since this attenuation was seen at 24h of hyperoxia, an early time point when hyperoxia-induced inflammatory response is still minimal, we surmised that ASK1 deletion-associated attenuation of hyperoxia-induced cellular apoptosis is through downregulation of downstream proapoptotic pathways of ASK1 in lung epithelial cells; thus, not through a blockade of secreted apoptosis-inducing cytokines from other cell types. At 48h hyperoxia (data not shown), the difference between TUNEL-stained lung sections from WT and ASK1 KO mice were minimal compared to 24h time point. A possible explanation for this inconsistent result is that prolonged exposure to hyperoxia caused other forms of cell death such as necrosis rather than apoptosis, making it difficult to clearly distinguish viable from non-viable cells by TUNEL staining.

The fate of alveolar macrophages under oxidative stress is not completely understood even though it is established that hyperoxic insult induces them to produce a variety of proinflammatory cytokines, growth factors, and chemoattractants. Previous reports revealed that macrophages are equipped with antioxidative defense mechanisms and can live for up to two years under a certain type of oxidative stress [27,28]. Our data indicates that under highly oxidative conditions, a part of alveolar macrophages undergo apoptosis and that the number of apoptotic macrophages in the lung airspace under oxidative stress is reduced in ASK1 KO mice. In the meantime, the current study does not reveal whether ASK1 KO-associated reduced number of apoptotic macrophages is due to increased resistance to oxidative stress or decreased recruitment of macrophage-committed mononuclear cells to the lung. Further investigation is required to answer this question.

ASK1 plays a critical role in the recruitment and activation of macrophages [29]. In this study, we found that ASK1 deletion not only hampers hyperoxia-induced apoptosis of lung epithelial cells and alveolar macrophages at early phase of HALI, but also suppresses an inflammatory response including neutrophil recruitment and proinflammatory cytokine elevation in the lung airspace. It remains to be elucidated however, if the suppression of airspace cytokine level in hyperoxic conditions seen in ASK1 KO mice is due to a reduction of cytokine release from alveolar macrophages, since other immune cell types or lung epithelial cells can be a source of secreted proinflammatory cytokines [30,31].

Our data suggest that the suppression of hyperoxia-induced cell apoptosis in lung tissue and air space is a direct effect of ASK1 deletion because apoptosis suppression is remarkable as early as 24 h of hyperoxia, an early time point in the course of HALI when proinflammatory cytokine elevation and immune cell recruitment is not distinguished. In the meantime, the hampered secretion of proinflammatory cytokines and immune cell recruitment to the lung seems to be an indirect effect of ASK1 deletion secondary to suppressed cell apoptosis. The most plausible reconciliation for these discrepant results is that ASK1 deletion leads to apoptosis suppression, which consequently obviates apoptosis-to-necrosis transition due to insufficient cleanup of apoptotic cells. As a result, this suppresses necrotic cell death-induced inflammatory response [32]. Taken together, these studies show that ASK1 KO mice have suppressed inflammatory response to HALI. The results demonstrate that activation of inflammatory cytokines and epithelial cell apoptosis in HALI is at least in part dependent on ASK1. Additionally, this study shows that deletion of the ASK1 gene protects against hyperoxia-induced accumulation of apoptotic macrophages in the lung airspace. Further studies are necessary to understand the precise mechanism of ASK1 in HALI. Clinically, inhibitors of ASK1 may be a novel approach to suppress hyperoxia-induced ALI and oxidant-mediated inflammatory diseases. Consequently, inhibitors of ASK1 may have potential therapeutic benefit in the management of ALI.

## Materials and Methods

### Ethical statement

All animal experiments were performed to comply with Institutional Animal Care and Use Committee (IACUC) of the University of South Florida for all surgical interventions. All animal experiments were conducted in accordance with the IACUC institutional guidelines. In addition, all the procedures that were performed on animals were in compliance with the approved IACUC protocol. For brochoalveolar lavage (BAL) fluid collection and mouse lung sample collection, mice were sedated with an intraperitoneal injection of ketmain/xylazine. Mice were euthanized immediately after hyperoxic exposure for sample collection. University of South Florida IACUC or ethics committee specifically approved this study.

### Mice

C57BL/6J mice (Harlan Laboratories, Indianapolis, IN) and ASK1 knockout (ASK1 KO) mice with the same genetic background (gift from Dr. Hidenori Ichijo, University of Tokyo, Tokyo, Japan) were used to conduct *in vivo* experiments. Mice, aged 7–9 weeks, were placed in cages in a chamber (75×50×50 cm) and exposed to 100% O_2_ for 24, 48 or 72 h. The oxygen concentration in the chamber was monitored and regulated with the proOx P100 sensor (BioSpherix, NY, USA). Mice were euthanized immediately after hyperoxic exposure for sample collection.

### Bronchoalveolar Lavage (BAL) fluid

BAL fluid was collected as described previously [6,14]. Briefly, mice were sedated with intraperitoneal (i.p.) injection of ketamine/xylazine. After cervical dislocation, a transverse incision was made on the ventral neck to expose the trachea. Then, a 0.6 mm catheter was placed into the trachea through the incision. To collect BAL fluid, we repeated the following procedure 3 times: 1 ml of cold sterile phosphate-buffered saline (PBS) was instilled into the lung using 1 ml syringe attached to the catheter, then BAL fluid was retrieved and added to collection tube. After the BAL fluid was centrifuged, the supernatants were stored at -80°C until further analysis.

### TUNEL staining on BAL fluid cells

BAL fluid (2 to 2.5 mL) was centrifuged at 400g for 10 min at 4°C. Cell pellets were resuspended in 1 ml of cold sterile PBS and total number of cells was immediately determined using hemocytometer. Two sets of aliquots, 200–300 μl, of cell suspension were centrifuged onto glass slides at 800 rpm for 3 min in a cytocentrifuge (Shandon Cytospin 2, Pittsburgh, PA). One set of cytospin cells was stained with Diff-Quik Stain and differential white blood cell count was performed. The other set of cytospun cells was fixed in 4% paraformaldehyde for 25 min at 4°C, stained with TdT-mediated dUTP nick end labeling (TUNEL) using DeadEnd Fluorometric TUNEL System (Promega, Madison, WI). Cells stained with TUNEL were counterstained with DAPI and fluorescence signals were detected on a BX-43 fluorescence microscope (Olympus, Tokyo, Japan). The number of cells positive for TUNEL and DAPI was counted respectively in 4 randomly chosen fields. For each field the ratio of cells positive for TUNEL to those positive for DAPI was calculated as an apoptotic ratio. The mean of the apoptotic ratios of 4 randomly chosen fields was used as the representative apoptotic ratio for each sample, which was multiplied by the density of total white blood cells to retrieve the density of apoptotic cells.

### Collection of Mouse Lung

Mice were anesthetized with intraperitoneal injection of a ketamine/xylazine mixture. Following thoracotomy, the inferior vena cava (IVC) was clamped and 2 ml of sterile PBS was injected into the right ventricle for lung perfusion. The whole lung was inflation-fixed in 10% neutralized formalin at 20 cm H_2_O pressure. Paraffin-embedded tissue sections were cut by microtome and stored at room temperature until use.

### ELISA

The levels of IL-1β (eBioscience, San Diego, CA) and TNF-α (RayBiotech Inc., Norcross, GA) in BAL fluid were measured using commercial ELISA kits according to the manufacturer's instructions.

### TUNEL staining on lung tissue sections

TUNEL staining of lung tissue sections was performed as previously described [6]. Briefly, the lung tissue sections were deparaffinized and then incubated with proteinase K to unmask antigen binding sites. After being blocked with 3% hydrogen peroxide in PBS, the sections were incubated with TdT enzyme and FITC-labeled dUTP. Subsequently, the sections were incubated with anti-FITC horseradish peroxidase conjugate. Colorimetric detection of apoptotic cells was performed using 3,3′-diaminobenzidine (DAB) reagent.

### Statistical analysis

Statistical analysis was performed with GraphPad Prism 5 (GraphPad Software version 10.0, San Diego, CA). Comparison of variables between two groups was performed using the Student's t-test. Comparison of variables between three and more groups was performed using one-way ANOVA with post-hoc Tukey HSD test. All tests were two-tailed, and values of p < 0.05 were considered significant.

## Supporting Information

S1 Table Density of immune cells and concentration of cytokines in WT and ASK1 KO mice exposed to room air and hyperoxia. WT and ASK1 KO mice were exposed to normoxia, or 100% O_2_ for 24, 48 or 72 h. The densities of each immune cell type in BAL fluid and the concentrations of IL-1β and TNF-α in supernatants of BAL fluid were determined. Results (means ± SEM; n = 3–6 in each group) are shown.
(TIF)

## References

[1] Johnson ER, Matthay MA. Acute lung injury: epidemiology, pathogenesis, and treatment. J Aerosol Med Pulm Drug Deliv. 2010;23(4):243–52. 10.1089/jamp.2009.0775 20073554 PMC3133560

[2] Cox RR Jr, Phillips O, Kolliputi N. Putting the brakes on acute lung injury: can resolvins suppress acute lung injury? Front Physiol. 2012;3:445 10.3389/fphys.2012.00445 23226129 PMC3509342

[3] Lagishetty V, Parthasarathy PT, Phillips O, Fukumoto J, Cho Y, Fukumoto I, et al Dysregulation of CLOCK gene expression in hyperoxia-induced lung injury. Am J Physiol Cell Physiol. 2014;306(11):C999–C1007. 10.1152/ajpcell.00064.2013 24696144 PMC4042094

[4] Cox R Jr, Phillips O, Fukumoto J, Fukumoto I, Tamarapu Parthasarathy P, Arias S, et al Aspirin-Triggered Resolvin D1 Treatment Enhances Resolution of Hyperoxic Acute Lung Injury. Am J Respir Cell Mol Biol. 2015. 10.1165/rcmb.2014-0339OC PMC4566067 25647402

[5] Matute-Bello G, Frevert CW, Martin TR. Animal models of acute lung injury. Am J Physiol Lung Cell Mol Physiol. 2008;295(3):L379–99. 10.1152/ajplung.00010.2008 18621912 PMC2536793

[6] Fukumoto J, Fukumoto I, Parthasarathy PT, Cox R, Huynh B, Ramanathan GK, et al NLRP3 Deletion Protects from Hyperoxia-Induced Acute Lung Injury. Am J Physiol Cell Physiol. 2013 Epub 2013/05/03. 10.1152/ajpcell.00086.2013 . 23636457 PMC3725631

[7] Makena PS, Gorantla VK, Ghosh MC, Bezawada L, Kandasamy K, Balazs L, et al Deletion of apoptosis signal-regulating kinase-1 prevents ventilator-induced lung injury in mice. Am J Respir Cell Mol Biol. 2012;46(4):461–9. 10.1165/rcmb.2011-0234OC 22052879 PMC3359950

[8] Hayakawa R, Hayakawa T, Takeda K, Ichijo H. Therapeutic targets in the ASK1-dependent stress signaling pathways. Proc Jpn Acad Ser B Phys Biol Sci. 2012;88(8):434–53. 23060232 10.2183/pjab.88.434 PMC3491083

[9] Liu Y, Min W. Thioredoxin promotes ASK1 ubiquitination and degradation to inhibit ASK1-mediated apoptosis in a redox activity-independent manner. Circ Res. 2002;90(12):1259–66. . 12089063 10.1161/01.res.0000022160.64355.62

[10] Yamada T, Iwasaki Y, Nagata K, Fushiki S, Nakamura H, Marunaka Y, et al Thioredoxin-1 protects against hyperoxia-induced apoptosis in cells of the alveolar walls. Pulm Pharmacol Ther. 2007;20(6):650–9. 10.1016/j.pupt.2006.07.004 . 17045827

[11] Kolliputi N, Waxman AB. IL-6 cytoprotection in hyperoxic acute lung injury occurs via suppressor of cytokine signaling-1-induced apoptosis signal-regulating kinase-1 degradation. Am J Respir Cell Mol Biol. 2009;40(3):314–24. 10.1165/rcmb.2007-0287OC 18776134 PMC2645529

[12] Matsuzawa A, Saegusa K, Noguchi T, Sadamitsu C, Nishitoh H, Nagai S, et al ROS-dependent activation of the TRAF6-ASK1-p38 pathway is selectively required for TLR4-mediated innate immunity. Nat Immunol. 2005;6(6):587–92. 10.1038/ni1200 . 15864310

[13] Galam L, Parthasarathy PT, Cho Y, Cho SH, Lee YC, Lockey RF, et al Adenovirus-mediated transfer of the SOCS-1 gene to mouse lung confers protection against hyperoxic acute lung injury. Free Radic Biol Med. 2015;84:196–205. 10.1016/j.freeradbiomed.2015.03.036 25850028 PMC4457693

[14] Ward NS, Waxman AB, Homer RJ, Mantell LL, Einarsson O, Du Y, et al Interleukin-6-induced protection in hyperoxic acute lung injury. Am J Respir Cell Mol Biol. 2000;22(5):535–42. . 10783124 10.1165/ajrcmb.22.5.3808

[15] Zaher TE, Miller EJ, Morrow DM, Javdan M, Mantell LL. Hyperoxia-induced signal transduction pathways in pulmonary epithelial cells. Free Radic Biol Med. 2007;42(7):897–908. 10.1016/j.freeradbiomed.2007.01.021 17349918 PMC1876680

[16] Savill J. Recognition and phagocytosis of cells undergoing apoptosis. Br Med Bull. 1997;53(3):491–508. . 9374033 10.1093/oxfordjournals.bmb.a011626

[17] Ganter MT, Roux J, Miyazawa B, Howard M, Frank JA, Su G, et al Interleukin-1beta causes acute lung injury via alphavbeta5 and alphavbeta6 integrin-dependent mechanisms. Circ Res. 2008;102(7):804–12. 10.1161/circresaha.107.161067 18276918 PMC2739091

[18] Kolliputi N, Shaik RS, Waxman AB. The inflammasome mediates hyperoxia-induced alveolar cell permeability. J Immunol. 2010;184(10):5819–26. 10.4049/jimmunol.0902766 . 20375306 PMC3780794

[19] Kolliputi N, Galam L, Parthasarathy PT, Tipparaju SM, Lockey RF. NALP-3 inflammasome silencing attenuates ceramide-induced transepithelial permeability. J Cell Physiol. 2012;227(9):3310–6. 10.1002/jcp.24026 22169929 PMC3323724

[20] Mukhopadhyay S, Hoidal JR, Mukherjee TK. Role of TNFalpha in pulmonary pathophysiology. Respir Res. 2006;7:125 10.1186/1465-9921-7-125 17034639 PMC1613248

[21] Noguchi T. ROS-dependent Activation of ASK1 in Inflammatory Signaling. Journal of Oral Biosciences. 2008;50(2):107–14.

[22] Mo JS, Yoon JH, Ann EJ, Ahn JS, Baek HJ, Lee HJ, et al Notch1 modulates oxidative stress induced cell death through suppression of apoptosis signal-regulating kinase 1. Proc Natl Acad Sci U S A. 2013;110(17):6865–70. 10.1073/pnas.1209078110 23569274 PMC3637772

[23] Nakagawa H, Maeda S. Inflammation- and stress-related signaling pathways in hepatocarcinogenesis. World J Gastroenterol. 2012;18(31):4071–81. 10.3748/wjg.v18.i31.4071 22919237 PMC3422785

[24] Watanabe T, Otsu K, Takeda T, Yamaguchi O, Hikoso S, Kashiwase K, et al Apoptosis signal-regulating kinase 1 is involved not only in apoptosis but also in non-apoptotic cardiomyocyte death. Biochem Biophys Res Commun. 2005;333(2):562–7. 10.1016/j.bbrc.2005.05.151 . 15953587

[25] Martin TR, Hagimoto N, Nakamura M, Matute-Bello G. Apoptosis and epithelial injury in the lungs. Proc Am Thorac Soc. 2005;2(3):214–20. 10.1513/pats.200504-031AC 16222040 PMC2713319

[26] Waxman AB, Kolliputi N. IL-6 protects against hyperoxia-induced mitochondrial damage via Bcl-2-induced Bak interactions with mitofusins. Am J Respir Cell Mol Biol. 2009;41(4):385–96. 10.1165/rcmb.2008-0302OC 19168699 PMC2746985

[27] Marques LJ, Teschler H, Guzman J, Costabel U. Smoker's lung transplanted to a nonsmoker. Long-term detection of smoker's macrophages. Am J Respir Crit Care Med. 1997;156(5):1700–2. 10.1164/ajrccm.156.5.9611052 . 9372697

[28] Nyunoya T, Monick MM, Powers LS, Yarovinsky TO, Hunninghake GW. Macrophages survive hyperoxia via prolonged ERK activation due to phosphatase down-regulation. J Biol Chem. 2005;280(28):26295–302. 10.1074/jbc.M500185200 . 15901735

[29] Osaka N, Takahashi T, Murakami S, Matsuzawa A, Noguchi T, Fujiwara T, et al ASK1-dependent recruitment and activation of macrophages induce hair growth in skin wounds. J Cell Biol. 2007;176(7):903–9. 10.1083/jcb.200611015 17389227 PMC2064076

[30] Crestani B, Cornillet P, Dehoux M, Rolland C, Guenounou M, Aubier M. Alveolar type II epithelial cells produce interleukin-6 in vitro and in vivo. Regulation by alveolar macrophage secretory products. J Clin Invest. 1994;94(2):731–40. 10.1172/jci117392 8040328 PMC296153

[31] Thorley AJ, Ford PA, Giembycz MA, Goldstraw P, Young A, Tetley TD. Differential regulation of cytokine release and leukocyte migration by lipopolysaccharide-stimulated primary human lung alveolar type II epithelial cells and macrophages. J Immunol. 2007;178(1):463–73. . 17182585 10.4049/jimmunol.178.1.463

[32] Scaffidi P, Misteli T, Bianchi ME. Release of chromatin protein HMGB1 by necrotic cells triggers inflammation. Nature. 2002;418(6894):191–5. 10.1038/nature00858 . 12110890

