# Supplementary figures and images for: Deletion of ASK1 Protects against Hyperoxia-Induced Acute Lung Injury
Source: PLoS One. 2016 Jan 25;11(1):e0147652. doi: 10.1371/journal.pone.0147652 (PMC4726536; doi:10.1371/journal.pone.0147652)

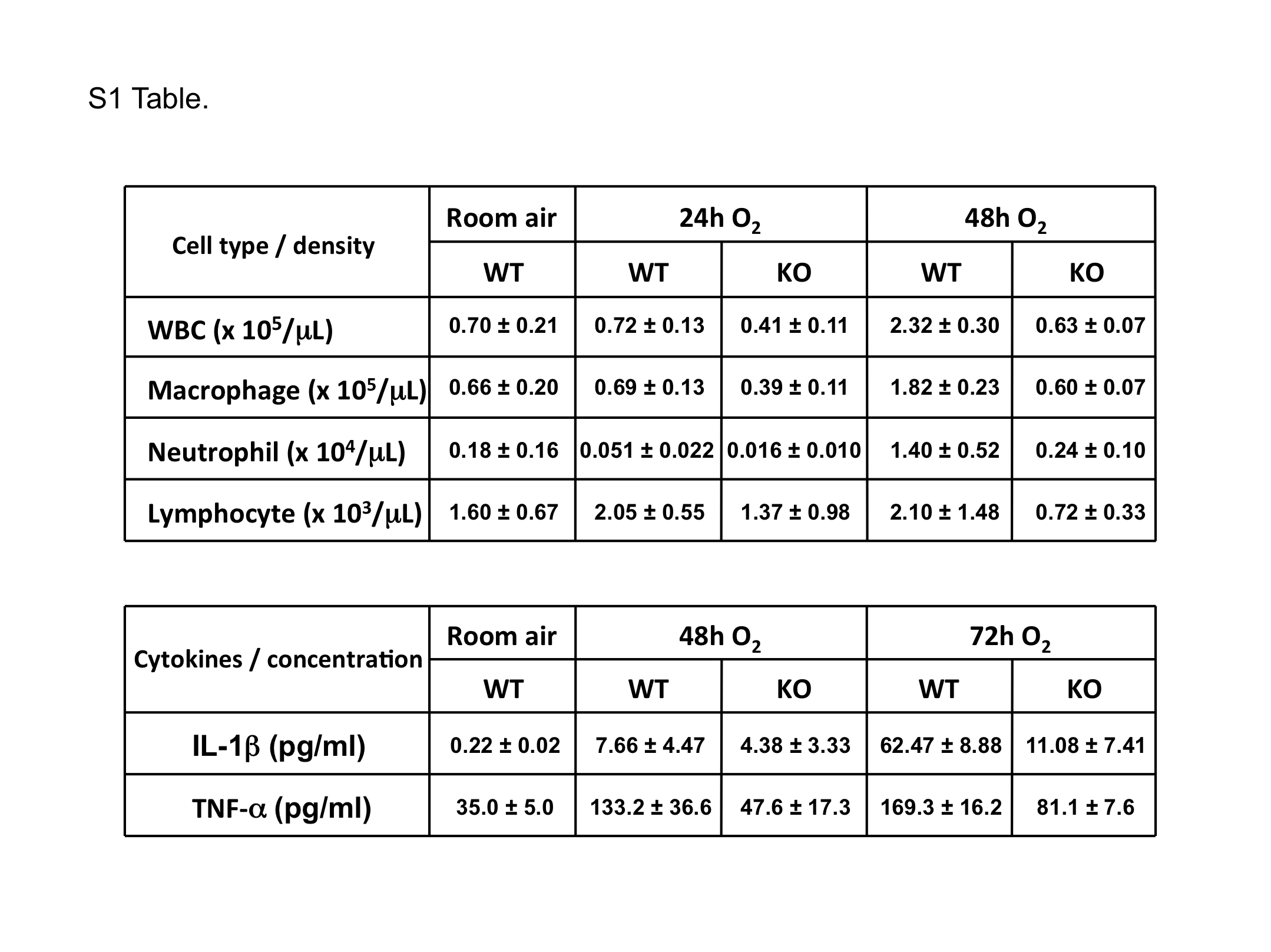

Supplement: S1 Table — WT and ASK1 KO mice were exposed to normoxia, or 100% O2 for 24, 48 or 72 h. The densities of each immune cell type in BAL fluid and the concentrations of IL-1β and TNF-α in supernatants of BAL fluid were determined. Results (means ± SEM; n = 3–6 in each group) are shown. (TIF) [file pone.0147652.s001.tif]
